# Magnetic Field-Driven Regulation of Bioactive Metabolites and Metabolic Enzyme Inhibition in *Sanghuangporus vaninii*

**DOI:** 10.3390/antiox15040406

**Published:** 2026-03-24

**Authors:** Qiurui Ma, Seo Yoon Lee, Zi Liu, Shuo Zhang, Jing Wang, KH Ahammad Uz Zaman, Helong Bai, Ki Hyun Kim

**Affiliations:** 1 College of Chemistry, Changchun Normal University, Changchun 130032, China; 190419040119@stu.ccsfu.edu.cn (Q.M.); 2309010213@stu.ccsfu.edu.cn (Z.L.); 210414020338@stu.ccsfu.edu.cn (S.Z.); wangjing018@ccsfu.edu.cn (J.W.); 2 School of Pharmacy, Sungkyunkwan University, Suwon 16419, Republic of Korea; allag8201@g.skku.edu; 3 DKI College of Pharmacy, University of Hawaii at Hilo, Hilo, HI 96720, USA; kzaman@hawaii.edu

**Keywords:** Static magnetic field, *Sanghuangporus vaninii*, secondary metabolites, chronic metabolic diseases

## Abstract

The effects of static magnetic field (SMF) treatment on the solid-state culture of *Sanghuangporus vaninii* (SV) were investigated to enhance metabolite production and bioactivity. SMF parameters including intensity, exposure duration, and temperature were optimized, and treatment at 4 mT for 2 h per day produced the most pronounced effects, increasing total flavonoid (TFC), polyphenol (TPC), and triterpenoid (TTC) contents by 61–438% compared with the control. Ultrasonic extraction and semi-preparative chromatography enabled the isolation of three key compounds: D-(+)-trehalose (**1**), 5,7-dihydroxy-3,4′-dimethoxyflavone (**2**), and pinolenic acid (**3**), all of which were elevated following SMF treatment. Importantly, SMF exposure was associated with enhanced inhibitory activities against enzymes relevant to chronic metabolic disorders. The overall inhibitory activities against α-amylase, α-glucosidase, pancreatic lipase, and xanthine oxidase increased by 6–28% compared with the control, reaching a maximum inhibition of 97.60 ± 0.17%. Preliminary in vitro screening at 100 μg/mL showed that compounds **1** and **2** inhibited both α-amylase and α-glucosidase, whereas compound **3** selectively inhibited pancreatic lipase. Subsequent IC_50_ analysis confirmed that compound **2** under SMF treatment exhibited inhibitory activity comparable to acarbose against α-amylase (45.62 μg/mL vs. 52.18 μg/mL) and α-glucosidase (38.74 μg/mL vs. 35.42 μg/mL). In addition, compound **3** showed moderate inhibition of pancreatic lipase with an IC_50_ value of 42.15 μg/mL. These findings suggest that SMF treatment may enhance metabolite production and in vitro enzyme inhibitory activity in *S. vaninii*. However, these results are limited to in vitro assays, and further studies including cellular and in vivo validation, toxicity assessment, and pharmacokinetic evaluation, are required before any therapeutic or industrial applications can be considered.

## 1. Introduction

*Sanghuangporus vaninii* (SV), commonly known in Chinese as the “Sanghuang” mushroom and previously classified as *Phellinus vaninii* or *Inonotus vaninii*, is a perennial medicinal fungus that grows on both living and fallen poplar trees. Its fruiting bodies and cultured mycelia produce a diverse array of secondary metabolites, including flavonoids, terpenes, polyphenols, polysaccharides, and organic acids. Overall, pharmacological studies indicate that SV exhibits a wide range of bioactivities, including antitumor, immunomodulatory, hepatoprotective, anti-angiogenic, antibacterial, and antioxidant effects [1,2,3]. Although SV has shown promising therapeutic potential in the treatment of inflammatory conditions, relatively few studies have explored its effects on chronic metabolic diseases [4,5,6,7,8]. SV is both saprophytic and parasitic and grows very slowly in nature, resulting in a high market value. Consequently, laboratory cultivation has become a practical strategy for producing stable and abundant biomass. In this study, we evaluated the secondary metabolite content and enzyme inhibitory activities of SV to identify optimal culture conditions using both single-factor experiments and orthogonal experimental design.

The growth of organisms requires not only adequate nutrients but also optimal environmental conditions [9]. Factors such as temperature, culture duration, and physical stimuli have been shown to markedly influence microbial proliferation and metabolite production. For instance, temperature strongly affects the growth of *Xylaria nigripes*, thereby enhancing its biomass and the accumulation of bioactive compounds [10]. Similarly, regulation of the culture duration of *Monascus purpureus* has been found to significantly impact exopolysaccharide synthesis [11]. Among physical factors, magnetic fields (MFs) have recently attracted considerable attention due to their diverse biological effects [12,13,14,15,16,17,18]. MFs are generally classified into static magnetic fields (SMFs), low-frequency time-varying MFs, high-frequency MFs, and other specialized field types. In an SMF, magnetic intensity remains constant over time. Typically, MF intensity and exposure duration are considered key variables influencing biological responses. Several studies have demonstrated the biological impact of SMFs. Wheat seeds exposed to an SMF (100 mT or 200 mT) for 1 h exhibited substantially improved vitality indices after 16 h, indicating that SMF exposure can enhance seed vigor and promote growth [19]. Likewise, magnetic field treatment of soybean seeds has been reported to stimulate globulin production while inhibiting alcohol-soluble protein synthesis, ultimately increasing the levels of several fatty acids and improving soybean oil yield [20]. Exposure to a 60 mT magnetic field also increased cyanobacterial biomass by 37% compared with the control group [21]. In contrast, relatively high-intensity magnetic fields (30 mT and 40 mT) did not inhibit the growth of nano-green algae, whereas a lower-intensity field (20 mT) promoted growth [22]. MFs can also influence the transmembrane transport of charged particles, thereby affecting membrane signal transduction, fluidity, and the movement of substances across the cell membrane [23]. Moreover, exposure to high-intensity magnetic fields (200 mT) has been shown to alter the concentrations of potassium, calcium, and chloride ions, leading to changes in membrane potential and osmotic pressure [24].

Clinically, synthetic enzyme inhibitors such as allopurinol, acarbose, and orlistat are widely used to manage chronic metabolic disorders. Although these agents are effective and relatively inexpensive, their administration is frequently accompanied by undesirable side effects, including abdominal pain, bloating, and intestinal discomfort [25,26]. These limitations have increased interest in natural, non-toxic alternatives with comparable or improved efficacy. *S. vaninii* (SV) possesses diverse bioactive properties, and increasing evidence suggests that physical stimuli, such as magnetic fields, can enhance microbial metabolite production. Therefore, we hypothesized that exposure to a static magnetic field (SMF) could represent an effective strategy to improve the biochemical and functional properties of SV. Therefore, as part of our ongoing efforts to discover potential bioactive compounds [27,28,29,30], the present study aimed to optimize SV culture conditions under SMF treatment to enhance the production of bioactive secondary metabolites and improve the inhibitory activities of key metabolic enzymes, including α-amylase, α-glucosidase, pancreatic lipase, and xanthine oxidase. These findings provide a basis for further investigation of *S. vaninii* as a potential source of bioactive compounds.

## 2. Materials and Methods

### 2.1. Materials

**Figure 1:**
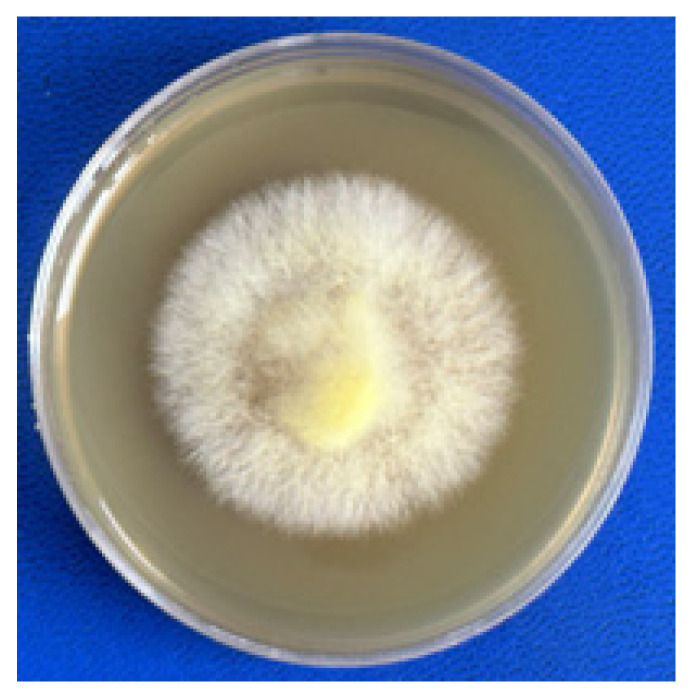
*Sanghuangporus vaninii* (SV) grown for 10 days on potato dextrose agar (PDA).

*Sanghuangporus vaninii* (SV) used in this study was provided by the Cultivation Base of Changchun Normal University (Figure 1). α-Amylase, α-glucosidase, pancreatic lipase, xanthine oxidase, DNS, PNPG, Na_2_CO_3_, 4-nitrophenyl laurate, and Tris–HCl buffer were purchased from Shanghai Yuanye Bio-Technology Co, Ltd. (Shanghai, China). Petroleum ether was obtained from Shanghai Aladdin Bio-Chem Technology Co., Ltd (Shanghai, China). The instruments used in this study included an Agilent 1100 HPLC system (Thermo Fisher Technologies, Waltham, MA, USA), a KH-250DB CNC ultrasonic cleaner (Kunshan Hechuang Ultrasonic Instrument Co., Ltd., Kunshan, China), an RE-52AA rotary evaporator (Shanghai Yarong Biochemical Instrument Factory, Shanghai, China), a CF312L-B condensing cycle device (Yamato Technology Trading, Shanghai Co., Ltd., Shanghai, China), and an MFC10 magnetic field incubator (Yinduste (Wuxi) Induction Technology Co., Ltd., Wuxi, Jiangsu, China).

### 2.2. Single-Factor Experiment

All static magnetic field (SMF) treatments were conducted using a magnetic field incubator (MFC10, Induce, Wuxi, China). The device generates a static magnetic field using built-in permanent magnets, with the field orientation adjustable to either longitudinal or transverse directions relative to the culture medium. Prior to the experiments, the magnetic flux density at the sample position was calibrated using a calibrated Gauss/Tesla meter. Field uniformity within the incubation chamber was verified by measuring the magnetic field intensity at nine evenly distributed positions (3 × 3 grid) within the sample placement area. The spatial variation in magnetic field intensity was confirmed to be within 5% of the nominal set value (e.g., 3.9–4.1 mT for a 4 mT setting). All incubations, including control experiments, were conducted in the same incubator under identical temperature conditions. For the control groups, the magnetic field generation system was deactivated so that the samples were exposed only to the Earth’s natural magnetic field (approximately 0.05 mT). The internal temperature of the incubator was continuously monitored throughout the experiments to ensure thermal stability and to exclude temperature-related effects as potential confounding factors.

#### 2.2.1. Selection of SMF Intensity

Fungi were placed in a static magnetic field (SMF) incubator (MFC10, Induce, China), and five SMF intensities were tested: 3 mT, 4 mT, 5 mT, 7 mT, and 10 mT. The culture temperature was maintained at 25 °C, and the cultivation period was set to 10 days. Total flavonoid content (TFC), total polyphenol content (TPC), and total triterpenoid content (TTC) in the crude SV extract were quantified, and the inhibitory activities of α-amylase, α-glucosidase, pancreatic lipase, and xanthine oxidase were evaluated.

#### 2.2.2. Selection of Culture Temperature

Fungi were cultured in a temperature-controlled incubator at 22, 25, 28, 31, or 34 °C for 10 days, under exposure only to the Earth’s natural magnetic field. TFC, TPC, and TTC in the crude SV extract were measured, and the inhibitory activities against α-amylase, α-glucosidase, pancreatic lipase, and xanthine oxidase were determined.

#### 2.2.3. Selection of Culture Time

Fungi were incubated for 4, 5, 7, 10, or 15 days at 25 °C under exposure only to the Earth’s magnetic field. TFC, TPC, and TTC in the crude SV extract were quantified, and the inhibition of α-amylase, α-glucosidase, pancreatic lipase, and xanthine oxidase was assessed.

#### 2.2.4. Selection of Magnetic Treatment Time

**Figure 2:**
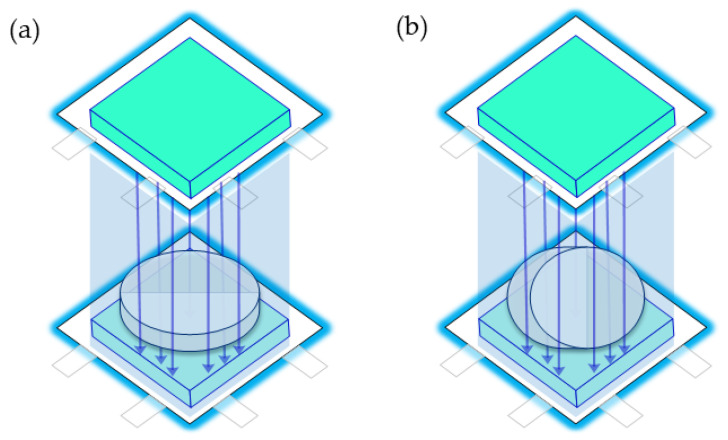
Schematic diagram of the SMF culture device. Arrows indicate the direction of the SMF. (**a**) Horizontal medium means that the medium acts in the longitudinal SMF; (**b**) Vertical medium means that the medium acts in the transverse SMF.

Fungi were placed in the SMF incubator and subjected to daily magnetic exposure for 1, 2, 6, 12, or 24 h. The SMF intensity was fixed at 4 mT, the temperature at 25 °C, and the total cultivation time at 10 days. The control group was cultured under identical conditions (temperature and duration) but was exposed only to the Earth’s magnetic field. TFC, TPC, and TTC in the crude SV extract were analyzed, and enzyme inhibitory activities against α-amylase, α-glucosidase, pancreatic lipase, and xanthine oxidase were evaluated. The SMF culture device is shown in Figure 2.

### 2.3. Orthogonal Experiment

Based on the results of the single-factor experiments, SPSS 27 software was used to analyze the effects of four factors: SMF intensity (A), culture temperature (B), culture time (C), and magnetic exposure time (D). An L_9_(3^4^) orthogonal design was employed, using TPC and the inhibition rate of xanthine oxidase as evaluation indicators to determine the optimal culture conditions. The factors and corresponding levels used in the orthogonal experiment are shown in Table 1. The orthogonal experimental design was analyzed to evaluate the relative effects of different experimental factors on the measured responses. Analysis of variance of the orthogonal test results was used to determine the optimal experimental conditions.

### 2.4. Cultivation of Sanghuangporus vaninii (SV) and Extract Preparation

Mycelial pieces of SV were activated by inoculation onto PDA medium and incubated at 28 °C until vigorous mycelial growth was observed. Under each culture condition, the SV samples were homogenized, extracted with methanol, and subjected to ultrasonic treatment for 1 h at 28 °C, repeated three times. The mixture was filtered, and the combined filtrates were concentrated under reduced pressure to obtain the crude SV extract.

### 2.5. Determination of Secondary Metabolites

#### 2.5.1. Total Flavonoid Content (TFC)

TFC was determined according to a previously reported method [31] with slight modifications. The crude extract was dissolved in 70% ethanol to a concentration of 1 mg/mL. For standard curve preparation, rutin standard solution was mixed with 5% NaNO_2_, 10% Al(NO_3_)_3_, and 4% NaOH, and the volume was adjusted with 70% ethanol. Absorbance was measured at 510 nm using a UV–Vis spectrophotometer. A standard curve was generated by plotting rutin concentration (mg/mL) against the absorbance values obtained from three independent replicate measurements.

#### 2.5.2. Total Polyphenol Content (TPC)

TPC was measured using the Folin–Ciocalteu colorimetric method as previously described [32]. Crude extract was dissolved in 70% ethanol (1 mg/mL). For standard curve construction, gallic acid solution was mixed with ultrapure water, followed by the addition of 10% Folin–Ciocalteu reagent and 10% Na_2_CO_3_. After incubation in the dark for 30 min, absorbance was measured at 760 nm using a UV–Vis spectrophotometer. A standard curve was generated by plotting gallic acid (mg/mL) concentration against the mean absorbance values from three independent replicate measurements.

#### 2.5.3. Total Triterpenoid Content (TTC)

TTC was determined based on a previously described method [33] with minor modifications. Crude extract was dissolved in 70% ethanol (1 mg/mL). For the standard curve, oleanolic acid was dissolved in chromatographic-grade methanol and sonicated. The solution was dried in a water bath at 75 °C, followed by sequential addition of 5% vanillin–glacial acetic acid, HClO_4_, and glacial acetic acid. Absorbance was measured at 550 nm using a UV–Vis spectrophotometer. A standard curve was generated by plotting oleanolic acid concentration (mg/mL) against the mean absorbance values from three independent replicate measurements, and the regression coefficient was calculated.

### 2.6. Determination of Enzyme Inhibition Rate

#### 2.6.1. α-Amylase

The α-amylase inhibition assay was performed following a modified version of the method reported by Apostolidis et al. [34]. The crude extract was dissolved in phosphate buffer (pH 6.8) and adjusted to a final concentration of 2 mg/mL. The reaction system is summarized in Table 2. All measurements were conducted in triplicate for each group.

#### 2.6.2. α-Glucosidase

The α-glucosidase inhibitory activity was evaluated using a modified version of a previously reported method [35]. The crude extract was dissolved in phosphate buffer (pH 6.8) and adjusted to a final concentration of 2 mg/mL. The reaction system was prepared according to the composition shown in Table 3. Each sample was analyzed in triplicate.

#### 2.6.3. Pancreatic Lipase

Pancreatic lipase inhibitory activity was evaluated according to a modified version of the method described by Kim et al. [36]. The crude extract was dissolved in Tris–HCl buffer (pH 8.2) and adjusted to a concentration of 2 mg/mL. The reaction system is presented in Table 4. All measurements were performed in triplicate.

#### 2.6.4. Xanthine Oxidase

Xanthine oxidase inhibitory activity was measured using a modified method based on Nile et al. [37]. The crude extract was dissolved in Tris–HCl buffer (pH 7.5) and adjusted to a final concentration of 2 mg/mL. The reaction setup is shown in Table 5. Each experiment was carried out in triplicate.

Inhibition rates of the crude SV extract were obtained using Equation (1):

(1) Inhibitionrate%=1−A1−A2A0−A3×100%

where ***A*_1_** is the absorbance of the sample, ***A*_2_** is the absorbance of the control group, ***A*****_0_** is the absorbance of the blank, and ***A*_3_** is the absorbance of the blank control.

### 2.7. Isolation of Major Metabolites from SMF-Treated SV

The crude extract obtained from the cultivation of SV under a 4 mT static magnetic field was suspended in water and partitioned with petroleum ether to remove nonpolar components. The petroleum ether layer was discarded, and the remaining aqueous fraction was subjected to preparative HPLC using a gradient elution of 10–100% MeOH/H_2_O over 30 min. The resulting fractions were further purified by semi-preparative HPLC to isolate individual compounds. Fraction A was purified by semi-preparative HPLC under isocratic conditions of 80% MeOH/H_2_O for 30 min, affording D-(+)-trehalose (**1**), 5,7-dihydroxy-3,4′-dimethoxyflavone (**2**), and pinolenic acid (**3**).

#### 2.7.1. D-(+)-Trehalose (**1**)

White powder; ESI-MS *m*/*z* 343.1 [M + H]^+^; ^1^H-NMR (600 MHz, CD_3_OD): *δ*_H_ 5.05 (1H, d, *J* = 3.8 Hz, H-1), 3.74 (3H, m, H-3, 6a, 6b), 3.60 (1H, dd, *J* = 12.2, 5.7 Hz, H-4), 3.41 (1H, dd, *J* = 9.7, 3.5 Hz, H-5), 3.26 (1H, m, H-2); ^13^C-NMR (150 MHz, CD_3_OD): *δ*_C_ 94.9 (C-1), 74.4 (C-3), 73.7 (C-2), 73.1 (C-5), 71.8 (C-4), 62.6 (C-6).

#### 2.7.2. 5,7-Dihydroxy-3,4′-dimethoxyflavone (**2**)

Yellow powder; ESI-MS *m*/*z* 317.1 [M + H]^+^; ^1^H-NMR (600 MHz, DMSO-d_6_): *δ*_H_ 12.67 (1H, s, 5-OH), 10.28 (1H, s, 7-OH), 7.98 (2H, d, *J* = 8.5 Hz, H-2′, 6′), 6.95 (2H, d, *J* = 8.5 Hz, H-3′, 5′), 6.74 (1H, d, *J* = 2.0 Hz, H-6), 6.37 (1H, d, *J* = 2.0 Hz, H-8), 3.86 (3H, s, 3-OCH_3_), 3.80 (3H, s, 4′-OCH_3_); ^13^C-NMR (150 MHz, DMSO-d_6_): *δ*_C_ 178.1 (C-4), 165.1 (C-7), 160.9 (C-9), 160.3 (C-4′), 156.3 (C-5), 155.9 (C-2), 137.8 (C-3), 130.2 (C-6′), 120.5 (C-1′), 115.6 (C-3′), 105.2 (C-10), 97.7 (C-6), 92.3 (C-8), 59.7 (3-OMe), 56.1 (4′-OMe).

#### 2.7.3. Pinolenic acid (**3**)

Light yellow liquid; ESI-MS *m*/*z* 279.2 [M + H]^+^; ^1^H-NMR (600 MHz, CD_3_OD): *δ*_H_ 5.42–5.33 (6H, m, H-5,6,9,10,12,13), 2.78 (2H, t, *J* = 6.2 Hz, H-11), 2.32 (2H, t, *J* = 7.8 Hz, H-2), 2.04–2.11 (8H, m, H-4,7,8,14), 1.69 (2H, quintet, *J* = 7.8 Hz, H-3), 1.25–1.40 (6H, m, H-15,16,17), 0.89 (3H, *J* = 7.6 Hz, H-18); ^13^C-NMR (150 MHz, CD_3_OD): *δ*_C_ 173.3 (C-1), 129.9 (C-13), 129.7 (C-6), 128.8 (C-9), 128.5 (C-5), 128.2 (C-10), 127.5 (C-12), 32.9 (C-2), 31.2 (C-16), 29.0 (C-15), 27.0 (C-8), 26.9 (C-7), 26.2 (C-4), 25.3 (C-14), 24.5 (C-11), 22.3 (C-3), 13.7 (C-18).

### 2.8. Determination of IC_50_ Values and Positive Control Experiments

For compounds that exhibited inhibitory activity at 100 μg/mL in the initial screening assay, half-maximal inhibitory concentration (IC_50_) values were determined using serial dilutions. Each compound was prepared at seven concentrations ranging from 10 to 500 μg/mL in the appropriate assay buffer (phosphate buffer for α-amylase and α-glucosidase assays; Tris–HCl buffer for pancreatic lipase assays). The enzyme inhibition assays were conducted as described in Section 2.6.1, Section 2.6.2, Section 2.6.3 and Section 2.6.4. The inhibition rate at each concentration was calculated using Equation (1). IC_50_ values were determined by plotting the percentage inhibition against the logarithm of compound concentration and fitting the data using nonlinear regression analysis. All experiments were performed in triplicate, and the results are expressed as mean ± standard deviation (SD).

To benchmark the inhibitory potency of the isolated compounds, standard inhibitors were used as positive controls: acarbose for α-amylase and α-glucosidase, orlistat for pancreatic lipase, and allopurinol for α-amylase. Each positive control was tested under the same experimental conditions and across the same concentration range as the tested compounds. IC_50_ values for the positive controls were calculated using the same method described above.

### 2.9. Statistical Analysis

All experiments were performed using three independent biological replicates (n = 3), and the results are expressed as mean ± standard deviation (SD). Statistical analyses were performed using SPSS software (IBM Corp., Armonk, NY, USA). Differences among multiple groups were evaluated using one-way analysis of variance (ANOVA), followed by Tukey’s post hoc multiple comparison test. Differences were considered statistically significant at *p* < 0.05.

## 3. Results

### 3.1. Standard Curves for Metabolite Quantification

**Figure 3:**
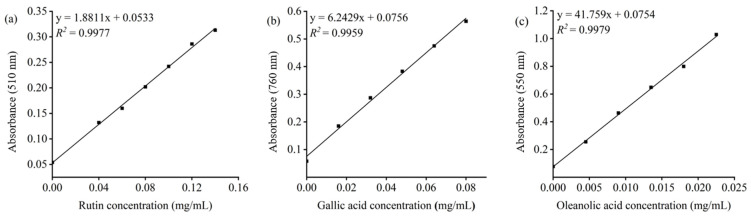
Standard curves of rutin (**a**), gallic acid (**b**), and oleanolic acid (**c**).

To determine the total flavonoid content (TFC), total polyphenol content (TPC), and total triterpenoid content (TTC), standard curves were constructed by plotting the concentrations of rutin (mg/mL), gallic acid (mg/mL), and oleanolic acid (mg/mL) against the corresponding absorbance values. As shown in Figure 3, all standard curves exhibited strong linearity (*R*^2^ > 0.99) and were subsequently used for the quantitative determination of TFC, TPC, and TTC in *Sanghuangporus vaninii* (SV).

### 3.2. Influence of SMF Intensity on Metabolite Content and Enzyme Inhibition

**Figure 4:**
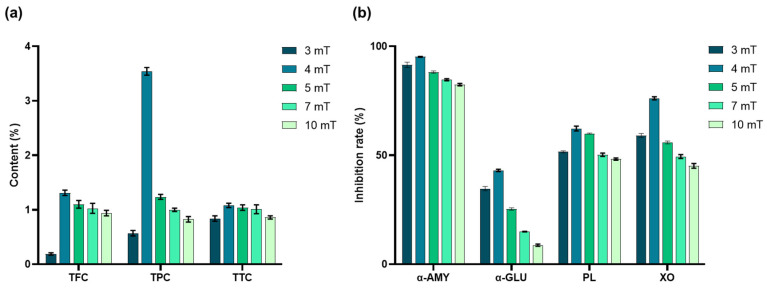
(**a**) Influence of SMF intensity on the content of secondary metabolites of SV. (**b**) Influence of SMF intensity on the inhibitory activities of α-amylase (α-AMY), α-glucosidase (α-GLU), pancreatic lipase (PL), and xanthine oxidase (XO) in SV.

Quantitative analysis revealed that the metabolite content of SV increased initially and then declined with increasing static magnetic field (SMF) intensity. At 4 mT, TFC, TPC, and TTC reached their highest values—1.31 ± 0.05%, 3.54 ± 0.07%, and 1.08 ± 0.04%, respectively (Figure 4a). Similarly, the inhibitory activities of α-amylase, α-glucosidase, pancreatic lipase, and xanthine oxidase also peaked at 4 mT, with inhibition rates of 95.19 ± 0.18%, 43.07 ± 0.53%, 62.28 ± 1.09%, and 76.16 ± 0.70%, respectively (Figure 4b). These results suggest that moderate SMF exposure promotes the accumulation of bioactive metabolites in *S. vaninii*. This increase is accompanied by enhanced inhibitory activity against metabolic enzymes.

### 3.3. Influence of Culture Temperature on Metabolite Content and Enzyme Inhibition

**Figure 5:**
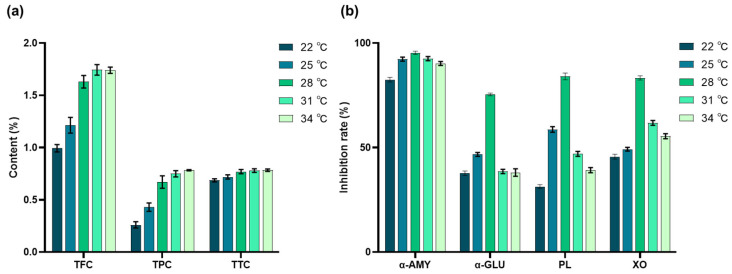
(**a**) Influence of culture temperature on the content of secondary metabolites of SV. (**b**) Influence of culture temperature on the inhibitory activities of α-amylase (α-AMY), α-glucosidase (α-GLU), pancreatic lipase (PL), and xanthine oxidase (XO) in SV.

Building upon the modulatory effects of SMF on metabolite production, temperature was next evaluated as an additional environmental factor influencing secondary metabolism in SV. TFC, TPC, and TTC gradually increased with rising temperature and reached stable levels at 28 °C (Figure 5a). Likewise, the inhibitory activities of α-amylase, α-glucosidase, pancreatic lipase, and xanthine oxidase (Figure 5b) first increased and then declined as the temperature continued to rise, with peak values observed at 28 °C (95.37 ± 0.79%, 75.45 ± 0.62%, 84.05 ± 1.61%, and 83.35 ± 0.99%, respectively). These findings suggest that 28 °C is the optimal temperature for SMF-treated SV to accumulate bioactive metabolites and exhibit maximal enzyme inhibitory activity.

### 3.4. Influence of Culture Time on Metabolite Content and Enzyme Inhibition

**Figure 6:**
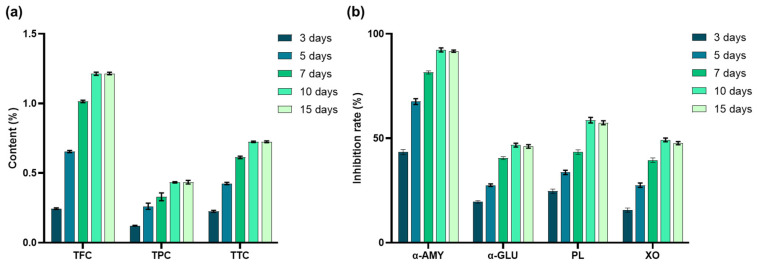
(**a**) Influence of culture time on the content of secondary metabolites of SV. (**b**) Influence of culture time on the inhibitory activities of α-amylase (α-AMY), α-glucosidase (α-GLU), pancreatic lipase (PL), and xanthine oxidase (XO) in SV.

Building on the findings from temperature optimization, we next examined the influence of culture duration on metabolite accumulation and enzyme inhibition in SV. Both the metabolite content and the enzyme inhibition rates of SV exhibited an overall upward trend with increasing culture time (Figure 6). Between 10 and 15 days, the increase in metabolite content slowed and gradually reached a steady state, after which 10 days was selected as the optimal culture period for subsequent experiments.

### 3.5. Influence of SMF Exposure Time on Metabolite Content and Enzyme Inhibition

**Figure 7:**
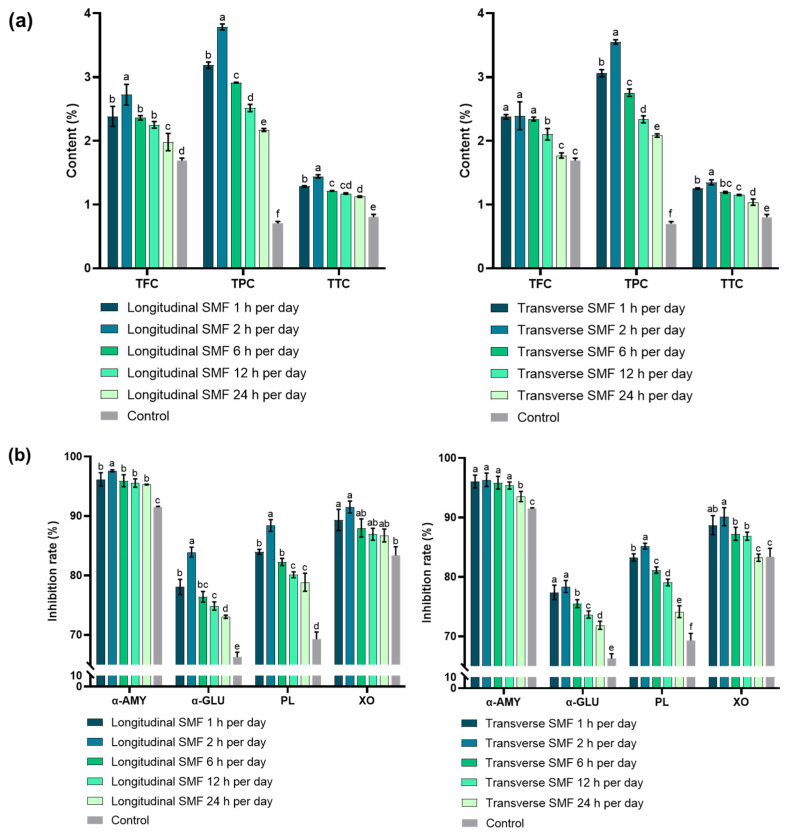
(**a**) Influence of magnetic treatment time on the content of secondary metabolites of SV. The different English letters show significance between each treatment while *p* < 0.05. (**b**) Influence of magnetic treatment time on the inhibitory activities α-amylase (α-AMY), α-glucosidase (α-GLU), pancreatic lipase (PL), and xanthine oxidase (XO) in SV. Different letters indicate statistically significant differences among treatments (*p* < 0.05).

Under a static magnetic field of 4 mT, the levels of TFC, TPC, and TTC reached their maximum after 2 h/day of longitudinal SMF exposure, yielding values of 2.72 ± 0.16%, 3.79 ± 0.05%, and 1.44 ± 0.03%, corresponding to increases of 61%, 438%, and 80% over the control group. After 2 h/day of transverse SMF exposure, TFC, TPC, and TTC increased by 41%, 404%, and 68%, respectively, relative to the control (Figure 7a). After 2 h/day of longitudinal SMF exposure, inhibition rates for α-amylase, α-glucosidase, pancreatic lipase, and xanthine oxidase reached 97.60 ± 0.17%, 83.89 ± 0.85%, 88.40 ± 0.99%, and 91.52 ± 1.00%, respectively. These values represent increases of 6%, 27%, 28%, and 10% compared to the control. Under transverse SMF exposure for 2 h/day, the corresponding increases were 5%, 18%, 23%, and 8%, respectively (Figure 7b).

### 3.6. Orthogonal Experiment on Optimization of Culture Conditions

These combined findings highlight the importance of evaluating the relative contribution of each cultivation factor to metabolite production and enzyme inhibition. As shown in Table 6, SMF intensity exerted the strongest influence on both metabolite content and enzyme inhibition rate, whereas temperature had the least effect. Under the optimized conditions, with an SMF intensity of 4 mT, a temperature of 28 °C, a culture duration of 10 days, and MF exposure for 2 h/day, TPC production reached its highest level (3.73%), and the inhibition rate of xanthine oxidase was also maximized (84.57%).

### 3.7. Comparison of Major Metabolites and Their Separation

**Figure 8:**
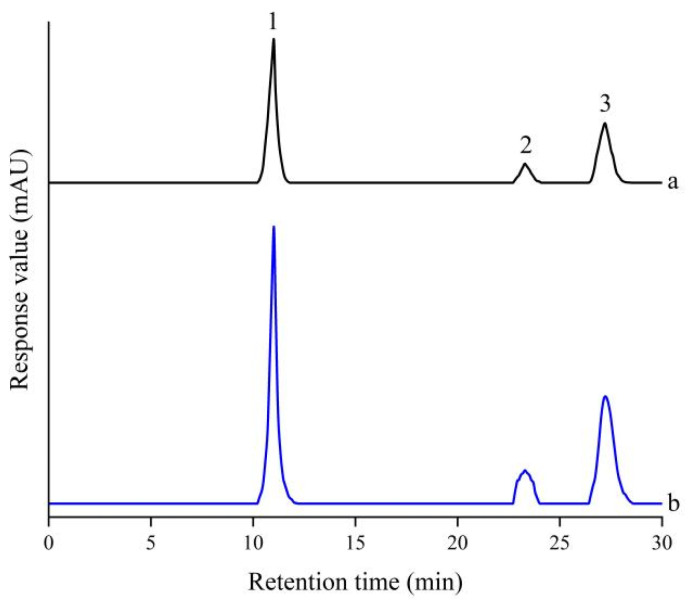
HPLC chromatogram of the aqueous fraction obtained after petroleum ether partition, used for the isolation of compounds **1**–**3**. (a) Culture of SV exposed to the Earth’s magnetic field; (b) Culture of SV treated with a static magnetic field.

Comparison of the HPLC chromatograms of various SV extracts led to the selection of three well-separated compounds as analytical targets. Notable differences in peak intensities among samples indicated substantial variation in the abundance of major constituents. When the chromatogram of the extract obtained under a 4 mT static magnetic field was compared with that of the extract cultivated under the Earth’s magnetic field, the retention times of the three target compounds were nearly identical, suggesting that the overall metabolite profiles of the two extracts were largely consistent. For further purification, the crude extract from SV cultures was suspended in water and partitioned with petroleum ether to remove nonpolar components. The petroleum ether layer was discarded, and the remaining aqueous fraction was subjected to HPLC analysis to compare the levels of the three target compounds. Semi-preparative HPLC analysis under isocratic conditions of 80% MeOH/H_2_O for 30 min revealed that the peak intensities of these metabolites were significantly higher in solid cultures treated with a 4 mT static magnetic field than in those exposed only to the Earth’s magnetic field (Figure 8).

**Figure 9:**
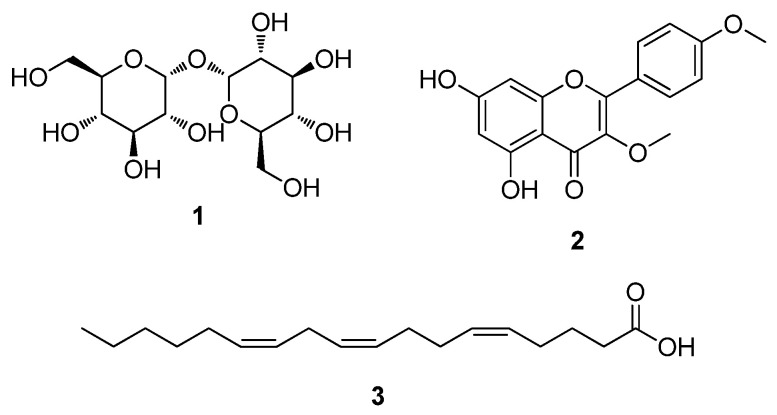
Chemical structures of the isolated compounds (**1**–**3**).

For structural elucidation, the aqueous fraction obtained after petroleum ether partition from the 4 mT treatment group was subjected to preparative and semi-preparative HPLC, yielding three major metabolites. These compounds (**1**–**3**) were identified as D-(+)-trehalose (**1**) [38], 5,7-dihydroxy-3,4′-dimethoxyflavone (**2**) [39], and pinolenic acid (**3**) [40,41] based on NMR spectroscopic and LC/MS analyses, as well as comparison with previously reported data in the literature (Figure 9).

### 3.8. Enzyme Inhibitory Activities of Isolated Compounds at 100 μg/mL

Compounds **1**–**3** were evaluated for their inhibitory activities against α-amylase, α-glucosidase, and pancreatic lipase, respectively. The results at a concentration of 100 μg/mL are presented in Table 7. At 100 μg/mL, compounds **1** and **2** exhibited significant inhibition of α-amylase. Further experiments showed that compound **2** treated with a 4 mT magnetic field inhibited α-amylase by 65.37 ± 0.05%, compared with 57.26 ± 0.11% for the untreated control. For compound **1**, the magnetic-field-treated version exhibited an inhibition rate of 68.61 ± 0.12%, whereas the untreated version showed 60.71 ± 0.06%. Both compounds **1** and **2** also significantly inhibited α-glucosidase at 100 μg/mL. Compound **2** treated with a 4 mT magnetic field showed an inhibition rate of 73.81 ± 0.12%, compared with 65.33 ± 0.14% for the untreated form. Likewise, compound **1** displayed an inhibition rate of 65.38 ± 0.09% after 4 mT treatment, whereas the untreated version exhibited 56.18 ± 0.02%. At 100 μg/mL, compound **3** exhibited a clear inhibitory effect on pancreatic lipase. The inhibition rate for compound **3** treated with a 4 mT magnetic field was 60.83 ± 0.03%, while the untreated version showed 53.77 ± 0.09%. Thus, the inhibition rate of compound **3** under 4 mT SMF treatment was significantly higher than that observed under geomagnetic field exposure in the solid cultures of *S. vaninii.*

### 3.9. Enzyme Inhibitory Activities and IC_50_ Values of Isolated Compounds

The inhibitory activities of compounds **1**–**3** against α-amylase, α-glucosidase, and pancreatic lipase were initially evaluated at a single concentration (100 μg/mL) and further characterized by determining their IC_50_ values. Standard enzyme inhibitors (acarbose, orlistat, and allopurinol) were included as positive controls for comparison (Table 8). As described in Section 3.8, compounds **1** and **2** showed inhibitory activity against α-amylase and α-glucosidase, whereas compound **3** selectively inhibited pancreatic lipase at 100 μg/mL (Table 7). For all three compounds, samples under 4 mT SMF treatment showed significantly higher inhibition rates than those under Earth’s magnetic field (Student’s *t*-test, *p* < 0.001). Based on these screening results, IC_50_ values were determined to further evaluate the inhibitory potency of the isolated compounds (Table 8). For α-amylase inhibition, compound **2** under 4 mT SMF treatment exhibited an IC_50_ value of 45.62 ± 1.87 μg/mL, which was significantly lower than that of the control group (Earth’s magnetic field) (67.89 ± 2.43 μg/mL, *p* < 0.01). Compound **1** showed IC_50_ values of 82.34 ± 3.21 μg/mL (SMF treatment) and 108.67 ± 4.53 μg/mL (control) (*p* < 0.05). The IC_50_ value of the positive control acarbose was 52.18 ± 2.06 μg/mL. For α-glucosidase inhibition, compound **2** under 4 mT SMF treatment showed an IC_50_ value of 38.74 ± 1.56 μg/mL, which was lower than that of the control group (58.91 ± 2.12 μg/mL, *p* < 0.01). Compound **1** exhibited IC_50_ values of 76.58 ± 2.89 μg/mL (SMF treatment) and 112.43 ± 4.17 μg/mL (control) (*p* < 0.01). The positive control acarbose showed an IC_50_ value of 35.42 ± 1.43 μg/mL. For pancreatic lipase inhibition, compound **3** under 4 mT SMF treatment displayed an IC_50_ value of 42.15 ± 1.78 μg/mL, significantly lower than that of the control group (58.36 ± 2.24 μg/mL, *p* < 0.01). The positive control orlistat exhibited a markedly lower IC_50_ value (8.76 ± 0.54 μg/mL), consistent with its known strong inhibitory activity against pancreatic lipase.

## 4. Discussion

The single-factor experiments revealed that SMF intensity significantly influences both metabolite production and enzyme inhibitory activity in *S. vaninii*. As shown in Section 3.2, moderate SMF exposure (4 mT) was associated with increased accumulation of bioactive metabolites and enhanced enzyme inhibitory activity. Comparable magnetically induced effects have also been reported in other fungal and plant species. For example, *Morchella eximia* exhibited high sensitivity to magnetic field treatment, showing a pronounced increase in total triterpenoid content (TTC) at 2.5 mT. Transcriptomic analysis further revealed that the upregulation of genes involved in terpenoid backbone and steroid biosynthesis pathways—including *mvaK2*, *ERG26*, *ERG3*, *EBP*, *SOAT*, and *ERG1*—contributed to the enhanced TTC in *M. eximia* [42]. Similarly, SMF exposure has been shown to affect the composition of alcohols, ketones, and esters in mushrooms, with total ketone content increasing at 2 mT and total thioether content increasing at 8 mT compared with control samples [43]. In *Anthemis gilanica*, SMF treatment at 4 mT significantly increased TPC and TFC, likely due to enhanced activity of enzymes associated with polyphenol biosynthesis [44]. Collectively, these findings suggest that the regulatory effects of SMF on microbial growth and secondary metabolite accumulation depend on multiple variables, including organism type, SMF intensity, and exposure duration, and that no linear relationship exists between SMF intensity and metabolic response.

In traditional Chinese medicine, secondary metabolites such as flavonoids, phenolics, terpenoids, sugars, and alkaloids are recognized as important natural inhibitors of α-amylase and α-glucosidase [45,46]. The inhibitory effects of polyphenols on these enzymes depend on structural characteristics, including the number of hydroxyl groups, degree of polymerization, and patterns of glycosylation and methylation. Hydroxyl substitutions at the 3, 5, and 7 positions on the A and C rings of flavonoids have been reported to enhance α-glucosidase inhibition [47]. Similarly, black tea polyphenols suppress postprandial hyperglycemia by inhibiting pancreatic lipase activity, thereby reducing triglyceride absorption and resulting in lower hypertriglyceridemia [48]. *Perilla frutescens* extracts have also demonstrated strong anti–xanthine oxidase activity, with flavonoid glycosides such as scutellarin, luteolin, apigenin, and rosmarinic acid identified as major active constituents [49]. Furthermore, compounds including neferol-3-*O*-rutin, methyl-rosmarinic acid, apigenin, and 4′,5,7-trimethoxyflavonoids isolated from *P. frutescens* have been shown to act as potent xanthine oxidase inhibitors [50]. Therefore, the enhanced inhibitory effects of SV on α-amylase, α-glucosidase, pancreatic lipase, and xanthine oxidase observed in this study may be partly attributable to the increased accumulation of these secondary metabolites induced by static magnetic field (SMF) treatment.

A temperature of 28 °C was identified as optimal for SMF-treated *Sanghuangporus vaninii* to promote the accumulation of bioactive metabolites and achieve maximal enzyme inhibitory activity. Similar temperature-dependent patterns of secondary metabolite accumulation have been reported in other fungi and plants. For example, *Xylaria nigripes* mycelia showed significant increases in both total polyphenol content (TPC) and total triterpenoid content (TTC) at 25 °C, highlighting the important role of temperature in stimulating bioactive compound production [10]. Moreover, in tomato fruit, seven differentially expressed MYB transcription factors, particularly SlMYB91, SlMYB106, and SlMYB70, were strongly correlated with structural genes involved in flavonoid biosynthesis, and additional genes associated with ripening and quality were also influenced by lower temperatures [51]. Together, these observations indicate that temperature is a key regulator of secondary metabolism, which may explain why *S. vaninii* exhibited the highest levels of TFC, TPC, and TTC, as well as the strongest enzyme inhibitory activities, at 28 °C in the present study. Consistent with our results, culture temperature exerts a substantial influence on the biosynthesis of secondary metabolites and may contribute to enhancing SV’s resistance to environmental stresses and diseases.

The metabolite contents and enzyme inhibitory activities of *S. vaninii* showed an overall increasing trend with prolonged culture time. Similar patterns have been reported in other fungi; for example, the optimal culture duration has been reported to be 22 days for *Antrodia camphorata* [52] and 54 h for *Cordyceps pruinosa* [53]. These observations indicate that the optimal culture time varies significantly among fungal species and that prolonged cultivation generally promotes the accumulation of secondary metabolites. However, beyond a certain threshold, the biosynthesis of these metabolites tends to plateau, and further extension of the culture period may not yield additional increases. Thus, the time-dependent changes in secondary metabolite content likely contribute to the enhanced inhibition of enzymes associated with chronic metabolic diseases observed in SV.

An SMF exposure duration of 2 h/day was identified as optimal for enhancing secondary metabolite accumulation, with longitudinal exposure exerting a stronger effect than transverse exposure. Collectively, these findings indicate that 2 h/day of SMF exposure more effectively enhances enzyme inhibitory activity than other exposure durations, with longitudinal SMF showing superior performance. Similar effects have also been reported in other microorganisms. For example, transverse SMF exposure markedly increased chlorophyll-a content in cyanobacteria, yielding 162% and 135% increases after 1 h/day and 24 h/day of treatment, respectively [54]. In another study, microalgae exposed to a 5 mT electromagnetic field for 1 h/day produced 31.1% carbohydrate, significantly higher than the 24.9% observed in controls [55]. These observations suggest that short daily MF exposure is more effective than continuous 24 h/day treatment in promoting metabolite accumulation. Consistent with these findings, our results indicate that MF-induced changes in secondary metabolite levels likely contribute to the enhanced inhibition of enzymes associated with chronic metabolic diseases.

Although the orthogonal experimental design employed in this study allows evaluation of the main effects of individual factors, it is important to acknowledge that potential interaction effects among the four variables (SMF intensity, culture temperature, culture time, and magnetic exposure duration) were not systematically analyzed. Based on the range analysis (R values), SMF intensity (A) was identified as the dominant factor influencing both total polyphenol content (TPC) and xanthine oxidase inhibition, followed by magnetic exposure time (D) and culture time (C), whereas temperature (B) showed the least influence. However, because interaction effects were not included in the present design, possible synergistic or antagonistic relationships between factors cannot be excluded. For example, the combined influence of SMF intensity and exposure duration may play an important role in modulating fungal metabolic responses. A longer exposure period could potentially amplify the effect of moderate SMF intensity, whereas elevated temperature might counteract stress responses induced by magnetic field exposure. Future studies employing more comprehensive experimental strategies, such as full factorial designs or response surface methodology (RSM), would enable systematic evaluation of interaction effects and provide a more refined optimization of culture conditions for maximizing bioactive metabolite production in *S. vaninii*.

Regarding trehalose (compound **1**), it should be noted that this compound is a common disaccharide primarily recognized as an energy reserve and stress protectant in fungi. To ensure the reliability of the observed inhibitory activity, the purity of the isolated trehalose was verified by HPLC–MS analysis, confirming a purity of >95% with no detectable co-eluting impurities with potential bioactivity. In addition, possible assay interference was evaluated through control experiments using the DNS reagent in the absence of enzyme. No significant color development was observed, indicating that trehalose does not interfere with the colorimetric detection system under the assay conditions used.

As a primary metabolite, trehalose is fundamentally distinct from the secondary metabolites (flavonoids, polyphenols, and triterpenoids) discussed elsewhere in this study. While secondary metabolites are often species specific and associated with ecological interactions, trehalose is ubiquitously present in fungi and fulfills essential physiological functions in energy storage and stress adaptation. The identification of D-(+)-trehalose (**1**) as a major compound in SMF-treated *S. vaninii* therefore warrants discussion of its biological relevance. Trehalose is widely recognized as a key stress-associated metabolite in fungi, serving both as a reserve carbohydrate and as a protective molecule under adverse environmental conditions [56,57]. In response to heat, osmotic imbalance, oxidative stress, and other stressors, fungi rapidly accumulate trehalose to safeguard cellular structures. Mechanistically, trehalose stabilizes proteins and lipid membranes by replacing water molecules and forming hydrogen bonds with polar residues, thereby preventing denaturation and preserving structural integrity [58,59].

In the present study, static magnetic field (SMF) exposure may act as a mild physical stimulus capable of triggering stress-responsive metabolic adjustments in *S. vaninii*. A marked elevation in trehalose levels was observed under 4 mT SMF treatment, suggesting activation of adaptive metabolic pathways. However, direct molecular evidence (e.g., gene expression or enzyme activity analyses) would be required to conclusively verify this mechanism. Importantly, trehalose accumulation may indirectly support enhanced secondary metabolite production by maintaining cellular homeostasis and protecting metabolic enzymes involved in biosynthetic pathways. By stabilizing enzymatic systems under stress conditions, trehalose could facilitate sustained biosynthetic activity, thereby contributing to the increased levels of flavonoids, polyphenols, and triterpenoids documented in this study. Furthermore, trehalose itself has been reported to exhibit modest enzyme inhibitory activity [38], indicating that its direct contribution to the overall bioactivity of SV extracts cannot be excluded.

To establish a quantitative relationship between the increased abundance of specific metabolites and the enhanced bioactivity of the crude extract, further analytical investigations are required. In particular, the absolute concentrations of compounds **1**–**3** in both SMF-treated and control extracts should be determined using validated quantitative methods such as HPLC–MS/MS with external calibration curves. In addition, activity reconstitution experiments could be performed by combining purified or synthetic standards of these compounds at ratios corresponding to their natural abundances in the extracts. The inhibitory activities of these reconstructed mixtures could then be compared with those of the crude extracts to evaluate the relative contribution of individual metabolites. Furthermore, multivariate statistical approaches, such as partial least squares regression (PLSR), integrating metabolomic profiling data with bioactivity measurements, could help identify which metabolites contribute most significantly to the observed functional enhancement. Such strategies would provide stronger evidence linking metabolite enrichment to biological activity and represent an important direction for future research on *S. vaninii* extracts.

While this study demonstrates that SMF treatment enhances metabolite accumulation and in vitro enzyme inhibitory activity in *S. vaninii*, several limitations should be acknowledged. The present work focused primarily on metabolite profiling and enzyme inhibition assays, and molecular or biochemical analyses were not conducted. In particular, measurements of reactive oxygen species (ROS), antioxidant capacity assays (e.g., DPPH or ABTS), and gene expression analysis of flavonoid or triterpenoid biosynthetic pathways (e.g., RT-qPCR) were not performed. Therefore, the mechanisms underlying SMF-induced metabolic changes remain unclear, and the possible involvement of oxidative stress responses or metabolic pathway regulation should be considered hypothetical at this stage. In addition, the bioactivity evaluation in this study was limited to in vitro enzyme inhibition assays. Further studies using cellular or in vivo models, along with toxicity and pharmacokinetic assessments, will be necessary to evaluate the biological relevance and safety of SMF-treated *S. vaninii* extracts and their active constituents. Future investigations integrating molecular, biochemical, and physiological analyses will be essential to clarify the mechanisms underlying SMF-mediated metabolic regulation.

## 5. Conclusions

In this study, *S. vaninii* was cultivated under a 4 mT static magnetic field (SMF), and three compounds were isolated from the solid-state culture: D-(+)-trehalose (**1**), 5,7-dihydroxy-3,4′-dimethoxyflavone (**2**), and pinolenic acid (**3**). Preliminary in vitro screening at 100 μg/mL showed that compounds **1** and **2** inhibited both α-amylase and α-glucosidase, whereas compound **3** exhibited inhibitory activity against pancreatic lipase. IC_50_ analysis further indicated that compound **2** under SMF treatment displayed inhibitory activity comparable to acarbose against α-amylase (45.62 μg/mL vs. 52.18 μg/mL) and α-glucosidase (38.74 μg/mL vs. 35.42 μg/mL). Compound **3** showed moderate inhibition of pancreatic lipase with an IC_50_ value of 42.15 μg/mL. Under optimized culture conditions (4 mT SMF intensity, 28 °C, 10 days of cultivation, and 2 h/day magnetic exposure), the contents of total flavonoids, polyphenols, and triterpenoids increased substantially, and the in vitro inhibitory activities against α-amylase, α-glucosidase, pancreatic lipase, and xanthine oxidase were enhanced. The isolated compounds under SMF treatment also exhibited higher inhibitory activities than those of control groups, with approximately 1.3–1.5-fold improvements in IC_50_ values. Overall, these results indicate that SMF treatment is associated with enhanced in vitro enzyme inhibitory potential of *S. vaninii* extracts and isolated compounds. The optimized cultivation conditions (4 mT, 28 °C, 10 days, and 2 h/day SMF exposure) may provide a practical approach for producing *S. vaninii* biomass with increased levels of bioactive metabolites. However, the current findings are limited to in vitro assays and do not support claims of therapeutic or industrial applicability at this stage. The mechanisms underlying SMF-induced metabolic changes remain unclear, and further studies involving molecular investigation, toxicity evaluation, pharmacokinetic analysis, and in vivo validation are required before potential applications can be considered. Nevertheless, this study provides preliminary evidence that SMF exposure may represent a useful strategy for enhancing the bioactive potential of this medicinal fungus.

## Figures and Tables

**Table 1:** Factors and levels used in orthogonal experimental design of L_9_(3^4^).

Level	A SMF Intensity (mT)	B Culture Temperature (°C)	C Culture Time (days)	D Magnetic Treatment Time (h Per day)
1	3	25	7	1
2	4	28	10	2
3	5	31	15	6

**Table 2:** The volume of reagents used in α-amylase reaction.

	Sample (μL)	Phosphate Buffer (pH 6.8) (μL)	α-Amylase (μL)	Starch Solution (μL)	DNS (μL)	Ultra-Pure Water (μL)
*A*_1_	50	0	50	50	100	1000
*A*_2_	50	50	0	50	100	1000
*A*_0_	0	50	50	50	100	1000
*A*_3_	0	100	0	50	100	1000

**Table 3:** The volume of reagents used in α-glucosidase reaction.

	Sample (μL)	Phosphate buffer (pH 6.8) (μL)	α-Glucosidase (μL)	PNPG (μL)	Na_2_CO_3_ (μL)
*A*_1_	50	0	50	100	100
*A*_2_	50	50	0	100	100
*A*_0_	0	50	50	100	100
*A*_3_	0	100	0	100	100

**Table 4:** The volume of reagents used in pancreatic lipase reaction.

	Sample (μL)	Tris-HCl Buffer (pH 8.2) (μL)	Pancreatic Lipase (μL)	4-Nitrophenyl Laurate (μL)
*A*_1_	50	350	150	450
*A*_2_	50	500	0	450
*A*_0_	0	400	150	450
*A*_3_	0	550	0	450

**Table 5:** The volume of reagents used in xanthine oxidase reaction.

	Sample (μL)	Tris-HCl Buffer (pH 7.5) (μL)	Xanthine Oxidase (μL)	Xanthine Solution (μL)
*A*_1_	50	0	50	100
*A*_2_	50	50	0	100
*A*_0_	0	50	50	100
*A*_3_	0	100	0	100

**Table 6:** Results of orthogonal experiment on optimization of culture conditions.

Group		A	B	C	D	y_1_ (TPC %)	y_2_ (Xanthine Oxidase Inhibition Rate %)
1		1	1	1	1	0.49	50.35
2		1	2	3	2	0.66	67.48
3		1	3	2	3	0.52	55.80
4		2	1	3	3	3.29	70.12
5		2	2	2	1	3.73	84.57
6		2	3	1	2	3.50	73.48
7		3	1	2	2	1.51	69.99
8		3	2	1	3	1.07	48.49
9		3	3	3	1	1.37	62.46
TPC	k_1_	0.56	1.77	1.69	1.86		
	k_2_	3.51	1.82	1.92	1.89		
	k_3_	1.31	1.79	1.77	1.62		
	R_1_	2.95	0.05	0.23	0.24		
Primary and secondary order							A > D > C > B
Optimal combination							A_2_B_2_C_2_D_2_
Xanthine oxidase	k_1_′	57.88	63.49	57.44	65.79		
	k_2_′	76.06	66.84	70.12	70.31		
	k_3_′	60.31	63.91	66.69	58.13		
	R_2_	15.75	2.93	3.43	7.66		
Primary and secondary order							A > D > C > B
Optimal combination							A_2_B_2_C_2_D_2_

**Table 7:** Screening results of activity of compounds **1**–**3**.

Compounds	Conditions	Inhibition Rate (%) at 100 μg/mL		
		*α*-Amylase	*α*-Glucosidase	Pancreatic Lipase
**1**	4 mT static magnetic field	68.61 ± 0.12	65.38 ± 0.09	——
	Earth magnetic field	60.71 ± 0.06	56.18 ± 0.02	——
**2**	4 mT static magnetic field	65.37 ± 0.05	73.81 ± 0.12	——
	Earth magnetic field	57.26 ± 0.11	65.33 ± 0.14	——
**3**	4 mT static magnetic field	——	——	60.83 ± 0.03
	Earth magnetic field	——	——	53.77 ± 0.09

**Table 8:** IC_50_ values of compounds **1**–**3** and positive controls against α-amylase, α-glucosidase, and pancreatic lipase ^a^.

Compounds	Conditions	IC_50_ (μg/mL)		
		*α*-Amylase	*α*-Glucosidase	Pancreatic Lipase
**1**	4 mT static magnetic field	82.34 ± 3.21	76.58 ± 2.89	>500
	Earth magnetic field	108.67 ± 4.53	112.43 ± 4.17	>500
**2**	4 mT static magnetic field	45.62 ± 1.87	38.74 ± 1.56	>500
	Earth magnetic field	67.89 ± 2.43	58.91 ± 2.12	>500
**3**	4 mT static magnetic field	>500	>500	42.15 ± 1.78
	Earth magnetic field	>500	>500	58.36 ± 2.24
**Acarbose** ^b^	——	52.18 ± 2.06	35.42 ± 1.43	NT
**Orlistat** ^b^	——	NT	NT	8.76 ± 0.54
**Allopurinol** ^b^	——	5.23 ± 0.31	NT	NT

^a^ Data are presented as mean ± standard deviation (SD) (n = 3). NT: not tested. >500 indicates less than 50% inhibition at the highest concentration tested (500 μg/mL); ^b^ Positive controls.

## Data Availability

The data presented in this study are available on request from the corresponding author. The data is not publicly available due to privacy restrictions.
